# Temperature Dependence of G and D’ Phonons in Monolayer to Few-Layer Graphene with Vacancies

**DOI:** 10.1186/s11671-020-03414-w

**Published:** 2020-09-30

**Authors:** Mingming Yang, Longlong Wang, Xiaofen Qiao, Yi Liu, Yufan Liu, Yafang Shi, Hongli Wu, Baolai Liang, Xiaoli Li, Xiaohui Zhao

**Affiliations:** 1 Hebei Key Laboratory of Optic-electronic Information and Materials, College of Physics Science & Technology, Hebei University, Baoding, 071002 People’s Republic of China; 2 State Key Laboratory of Superlattices and Microstructures, Institute of Semiconductors, Chinese Academy of Sciences, Beijing, 100083 People’s Republic of China

**Keywords:** Defects, Raman spectra, Temperature dependence, Thickness dependence

## Abstract

The defects into the hexagonal network of a sp^2^-hybridized carbon atom have been demonstrated to have a significant influence on intrinsic properties of graphene systems. In this paper, we presented a study of temperature-dependent Raman spectra of G peak and D’ band at low temperatures from 78 to 318 K in defective monolayer to few-layer graphene induced by ion C+ bombardment under the determination of vacancy uniformity. Defects lead to the increase of the negative temperature coefficient of G peak, with a value almost identical to that of D’ band. However, the variation of frequency and linewidth of G peak with layer number is contrary to D’ band. It derives from the related electron-phonon interaction in G and D’ phonon in the disorder-induced Raman scattering process. Our results are helpful to understand the mechanism of temperature-dependent phonons in graphene-based materials and provide valuable information on thermal properties of defects for the application of graphene-based devices.

## Introduction

Graphene-based materials have been promising materials bridging thermal, electronic, and photonic devices [1, 2] because of their intriguing properties [3, 4] since most studies were firstly focused on monolayer graphene (1LG) [3, 4] and subsequently transferred to few-layer graphenes (FLGs) [5, 6] due to their promising bandgap tunability [7, 8]. Raman scattering is one of the widely used techniques to characterize the phonon properties of graphene-based materials [2, 9]. Their thermal transport properties can be investigated by studying temperature-dependent (T-dependent) Raman spectra. Balandin et al. [10] first measured the thermal conductivity of a mechanically exfoliated 1LG by monitoring the shift of G peak with laser heating, and Ghosh et al. [11] subsequently investigated the thermal transport in mechanically exfoliated FLGs using the same technique. In many practical applications, defects in 1LG and FLGs are inevitable by different preparation methods and even modification of perfect graphene structures is required to tailor electrical parameters and to improve low chemical activity [12, 13]. It is indispensable to study how the defects affect the phonon properties of graphene to obtain an in-depth understanding of their thermal transport properties. Despite there have been few reports about T-dependent phonon properties in the case of nitrogen-doped and boron-doped graphene layer films [14], there has been no mechanism discussion because the potentially responsible mechanisms were relatively complex, such as the Fermi level change due to charge impurities, the N–C or B–C bond length change, and the long-range interactions between nitrogen or boron point defects. Up to now, there has been no report that specially investigates T-dependent phonon properties in graphene with vacancies. However, vacancies [15] are one of the most likely defects to occur in synthetic graphene materials with a one-atom-thick sheet of covalently bonded carbon atoms with sp^2^ hybridization packed in a honeycomb crystal lattice.

To clarify different phonon properties with pristine graphene, we performed a T-dependent Raman measurement of mechanically exfoliated 1LG and FLGs after ion C+ bombardment. Ion beam bombardment has been an effective method to finish graphene cutting and perforation [16], which can introduce vacancies with uniformity into the hexagonal network of carbon atoms by ion C+ bombardment. Besides the most important G peak (∼ 1582 cm^−1^) derived from intrinsic graphene structure, several additional symmetry breaking features near G peak such as the defect-related D’ peak [17] (∼ 1620 cm^−1^) can be found. In this paper, we presented a study of T-dependent phonon properties of G peak and D’ peak at low temperatures from 78 to 318 K in 1LG and FLGs with vacancies and tried to discuss the mechanism of the defective phonon effect and extrinsic T-dependent Raman behavior. Our results are helpful to provide T-dependent information of detects on thermal properties in graphene flakes for applications of devices.

## Materials and Methods

**Fig. 1:**
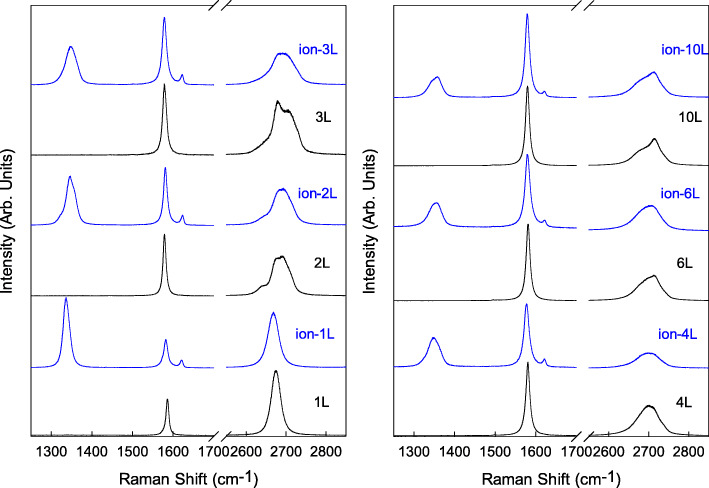
Raman spectra of 1LG-4LG, 6LG, and 10LG for defect-free and defective sets were measured at room temperature in the range of 1250–2850 cm^−1^

Highly oriented pyrolytic graphite (HOPG) was mechanically exfoliated on the same Si {100} substrates covered with an 89-nm SiO_2_ to obtain 1LG and FLGs. We used the notation NLG to indicate flakes with N layers. The layer number (*N*) of NLG was estimated by Raman measurements of the Si intensity ratio between the Si peak (*I*(Si_G_)) from SiO_2_/Si substrate overlying graphene flakes and the Si peak (*I*(Si_0_)) from bare SiO_2_/Si substrate [18]. The standard values of *I*(Si_G_)/*I*(Si_0_) for NLG flakes deposited on SiO_2_/Si substrate have been given in the supplementary data of reference [19]. We prepared several sets of graphene flakes with *N* determined and selected 2 sets of 1LG-4LG, 6LG, and 10LG flakes. Vacancies were introduced intentionally by ion C+ bombardment for one set of samples (called the defective set), with the defect-free set as a contrast. The low energy C+ ions bombarded perpendicularly to the sample surface at room temperature which was performed using an LC-4 type system with the dose and kinetic energy of 2 × 10^13^ cm^−2^ and 80 keV, respectively. After ion C+ bombardment, the D band at ∼ 1350 cm^−1^ and D’ peak at ∼ 1620 cm^−1^ appeared in the Raman spectra of NLG flakes, as depicted in Fig. 1. The Raman spectra of the defect-free set are also plotted in Fig. 1. Raman spectra were measured by the excitation of a 532-nm laser at room temperature under a × 100 objective lens (NA = 0.90). These two sets have the same thickness in order to facilitate the comparison. The G peak basically stayed at 1582 cm^−1^ before and after ion C+ bombardment, which showed that defects in the samples only broke the symmetry of carbon honeycomb lattice but did not cause evident doping which should make the frequency of G peak upshift. This made subsequent research more straightforward. There was another notable spectral band around 2700 cm^−1^ before and after ion C+ bombardment, which is referred to as a 2D band [17] and is an overtone of the D band [17]. The line shape of 2D band has been widely used to distinguish the number of graphene layers from one to four layers [20, 21]. However, the 2D band became mellow and full after ion C+ bombardment and its dependency on the number of graphene layers became blurred due to the lattice change to modify the phonon dispersion curve.

**Fig. 2:**
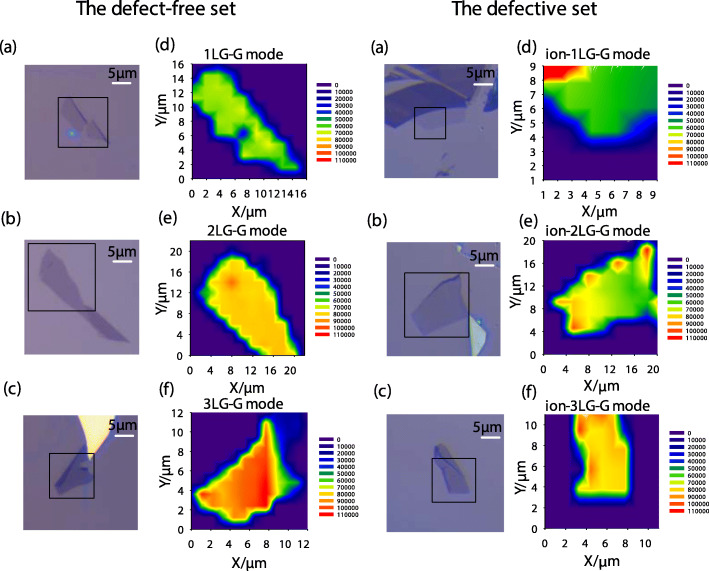
The mappings of *I*(G) for defect-free and defective 1LG, 2LG, and 3LG and the optical microscopic images of corresponding samples

In order to examine the uniformity of vacancies introduced in graphene structure by ion C+ bombardment, we measured Raman mapping of the samples from the defective set, with the defect-free set as a contrast. The Raman mappings were measured at room temperature in back-scattering with a HR Evolution micro-Raman system, equipped with the unique SWIFT™ CCD, a × 100 objective lens (NA = 0.90). An 1800 g/mm grating resulted in a 0.5-cm^−1^ spectral resolution. The laser excitation of 532 nm was used. A laser power below 2 mW was used to avoid sample heating. Mapping measurements were performed using a motorized stage. The *xy* coordinates of each point were previously set in order to find the optimized focus. Mapping images were constructed for each *xy* coordinate by taking 100 points on the surface of a sample with a 10 × 10 equally spaced array of probing points. In all cases *x*, *y* step was 0.5 μm. Raman spectra were measured in the range of 1250–2850 cm^−1^. The mappings of G peak intensity *I*(G) as a reference for defects contained in graphene flakes are shown in Fig. 2 for defect-free and defective 1LG, 2LG, and 3LG. The optical microscopic images of corresponding samples are also shown in Fig. 2. *I*(G) is sensitive to the number of defects [22] at low defect concentrations in graphene systems because G peak arises from the in-plane C–C bond stretching of all pairs of sp^2^ atoms in both rings and chains. Moreover, G peak is a phonon originating from a normal first-order Raman scattering process in graphene systems, and its intensity can be enhanced because of the resonance process [2] due to the excitation energy matching the transition from a valence band to a conduction band. The color of *I*(G) mappings in almost all samples is basically homogeneous over the entire sheet to determine the uniformity of the atomic structure of graphene layers. *I*(G) in defective NLG flakes is lower than that in defect-free NLG flakes due to the introduction of vacancies. Although the color of some points at the corner in the defective set of samples shows a bit difference, we can identify the uniformity of vacancies in the dominant part of defective samples. In addition, defects can be characterized by the average distance between nearest defects (*L*_D_) [22, 23]. We calculated the defect distribution *L*_D_ which is about 4–6 nm in C+-bombarded 1LG based on the intensity ratio between D band and G band, i.e., *I*(D)/*I*(G), using the well-known Tuinstra-Koenig relation [24] (the mapping of *L*_D_ in C+-bombarded 1LG was shown in Fig. f1 with more physical explanations in supplementary.) *I*(D) is also directly related with the number of defects [23, 25] because the D mode corresponds to a phonon due to the presence of defects. Considering that the D feature could be complex in FLGs [26] similar to the 2D band, the mappings of *I*(D) were shown for defective 1LG, 2LG, and 3LG in Fig. f2 of supplementary.

For the above prepared samples, we measured the T-dependent Raman spectra near G band (including G peak and D’ band) in both defect-free and defective sample sets of 1LG-4LG, 6LG, and 10LG flakes. The T-dependent Raman spectra were measured in back-scattering with a HR Evolution micro-Raman system, equipped with the unique SWIFT™ CCD. The samples were mounted on an in-house-made sample holder consisting of a thin copper disk with a central pillar and a 500-μm-diameter hole. Measurements were carried out in a liquid nitrogen (LN_2_) cooled low-temperature Linkam stage equipped with a temperature controller. The programmable cool-stage THMS600 (Linkam Scientific Instruments) covers the temperature range from 78 to 318 K in a N_2_ gas environment. The Linkam instrument has a temperature stability of ± 0.1 K. Using a grating with a groove density of 1800 g/mm, the achieved spectral resolution was 0.5 cm^−1^. A long working distance × 50 objective lens (NA = 0.45) was used, achieving a spatial resolution better than 1 μm. All spectra were excited with a 532-nm laser. During all the measurements, laser power has been kept low enough to prevent any sample heating. The integration time of 20 s was adopted to ensure a good signal-to-noise ratio. The T-dependence of Raman modes was measured in the range from 78 to 318 K and recorded at 10 K intervals, for the defect-free and defective sets.

## Results and Discussion

**Fig. 3:**
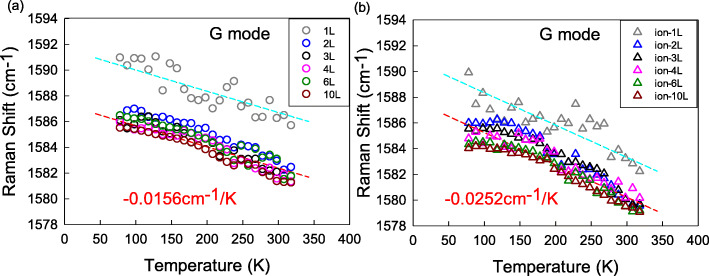
The T-dependent Pos(G) of 1LG-4LG, 6LG, and 10LG for **a** defect-free and **b** defective sets in the temperature range of 78–318 K

The studies are firstly concerned with the G peak. Figure 3 shows the T-dependent G peak position (Pos(G)) for the defect-free and defective sets. The data in 1LG are relatively fluctuating and away from the data of other layers. It is found that Pos(G) in both defect-free and defective 1LG shows a progressive downshift as the temperature increases, which indicates a linear relationship consistent with the reports for intrinsic graphene [14, 27, 28]. Pos(G) can be fitted to a linear equation, *ω*(*T*) = ω0 + *χT* [29], where *ω*0 is the peak position of vibrational bands at zero Kelvin temperature and *χ* represents the first-order temperature coefficient of the modes. The defect-free 1LG exhibits a negative temperature coefficient of − (1.56 ± 0.20) × 10^−2^ cm^−1^/K (plotted by the blue dotted line in Fig. 3a), which is basically consistent with the previous reports for intrinsic 1LG [14, 27, 28]. The temperature coefficient of the defective 1LG is found to be − (2.52 ± 0.20) × 10^−2^ cm^−1^/K (plotted by the blue dotted line in Fig. 3b), a value larger than that of the defect-free 1LG, similar with the previous reports of nitrogen doping or boron doping [14]. For samples with more layers, Pos(G) is considerably smaller than that of 1LG, but the T-dependent trend is approaching that of 1LG in both defect-free set (plotted by the pink dotted line in Fig. 3a) and defective set (plotted by the pink dotted line in Fig. 3b). Although some previous reports suggested that the temperature coefficient of the G peak in thicker samples is slightly smaller than that in 1LG [27, 28], our data show it is insensitive to the number of layers in the narrow range from 78 to 318 K. However, Pos(G) in the defect-free set are larger than those of the defective set, which should be a result of ion C+ bombardment.

**Fig. 4:**
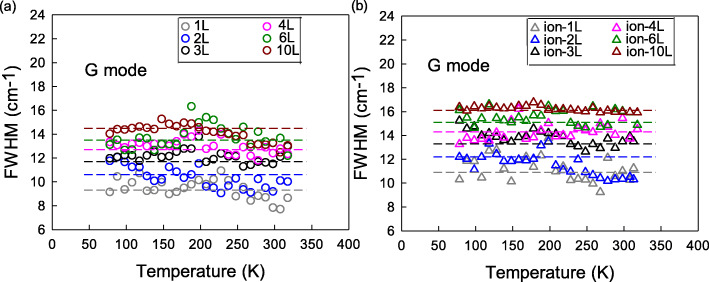
The T-dependent FWHM(G) of 1LG-4LG, 6LG, and 10LG for **a** defect-free and **b** defective sets in the temperature range of 78–318 K

Raman linewidth is another significant quality for uncovering the interactions of electrons and phonons when the crystal structure changes. Figure 4 shows the T-dependent full width at half maximum of G peak (FWHM(G)) for the defect-free and defective sets. It is found that FWHM(G) is not sensitive to temperature for both defect-free and defective sets, which is consistent with the recently reported T-dependent FWHM(G) results of pristine graphite [30]. It is interesting to note that T-dependent FWHM(G) in various graphene samples have been discussed [14, 31, 32] and have some discrepancies; for example, Lin et al. [31] observed an increase trend in unsupported graphene, Kolesov et al. [32] showed different T-dependencies in supported graphene on various substrates, and even Late et al. [14] showed slightly positive or insensitive dependencies in the case of nitrogen-doped or boron-doped graphite. However, in the low-temperature range below 350 K, FWHM(G) always kept constant in all the samples [14, 31, 32] probably due to weaker contribution from phonon anharmonicity and electron-phonon coupling (EPC) at low-temperature range [29, 33]. In addition, FWHM(G) from 1LG to 10LG is from 9.2 to 14.6 cm^−1^ in the defect-free set and from 10.9 to 16.1 cm^−1^ in the defective set. The FWHM(G) values in the defective set are larger than those in the defect-free set, which should be another result of ion C+ bombardment.

**Fig. 5:**
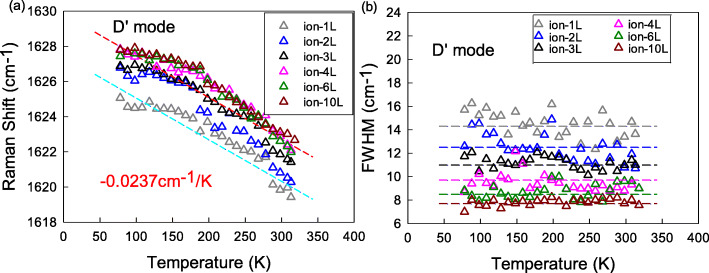
The T-dependent **a** Pos(D’) and **b** FWHM(D’) of 1LG-4LG, 6LG, and 10LG for the defective set in the temperature range of 78–318 K

We then studied the defect-related D’ band. Figure 5a shows Pos(D’) for the defective set. When the temperature increases from 78 to 318 K, Pos(D’) linearly decreases to 1620 cm^−1^ in C+ bombarded 1LG with a slope of around − (2.37 ± 0.20) × 10^−2^ cm^−1^/K (plotted by the blue dotted line in Fig. 5a). Pos(D’) shifts to larger values in thick layers but has a similar T-dependent slope approach to that of 1LG (plotted by the pink dotted line in Fig. 5a). FWHM(D’) shows no obvious T-dependence as shown in Fig. 5b. FWHM(D’) ranges from 7.6 to 14.4 cm^−1^ in 1LG to 10LG, but it decreases with increasing layers. It is obvious that D’ band shows a similar temperature coefficient with G peak after ion C+ bombardment. However, Pos(D’) increases whereas Pos(G) decreases; simultaneously, FWHM(D’) decreases whereas FWHM(G) increases as graphene layers become thicker.

By reviewing the previous work, we come to realize that there are several factors that influence the Raman spectra of the graphene systems. First, the T-dependent Raman study of pristine graphene has been explained by phonon anharmonicity and EPC [29]. However, the Raman spectra can be also dependent on the sample in the presence of vacancies. The temperature coefficient of G peak in defective graphene samples is found to be larger than that of the defect-free samples. Because EPC induces the increase of Pos(G) whereas phonon anharmonicity decreases it when the temperature increases, the domination of phonon anharmonicity leads to the softening of G phonon and hence results in a negative temperature coefficient for G peak [29]. After ion C+ bombardment, it is possible that the lattice change modifies the EPC leading to the hardening of G phonon; correspondingly, the temperature coefficient of G peak becomes less negative. Meanwhile, Pos(G) in the defect-free set are larger than those of the defective set, which means a decrease in the phonon energy due to the lattice change by vacancies [34]. Second, the FWHM(G) values in the defective set are larger than those in the defect-free set, which means a decrease in the phonon lifetime because of the phonon confinement effect [35] when the atom structure of graphene is destroyed by vacancies. Third, FLGs are formed by stacking numbers of 1LG along the c-axis, and their phonon anharmonicity and EPC are closely related to that of 1LG. The temperature coefficient of G band in FLGs is approaching that of 1LG in both defect-free and defective samples. However, there are some differences between them. The ultrathin nature of 1LG makes it necessary to consider the effect of the substrate. Pos(G) in 1LG is higher than that of the thicker samples for both defect-free and defective sets. Pos(G) shifts up to ~ 1588 cm^−1^ in defect-free 1LG and ~ 1584 cm^−1^ in defective 1LG at 300 K in variable temperature experiments although their Pos(G) basically stay at 1582 cm^−1^ in room temperature measurements. The possible reason is the thermal expansion coefficient mismatch between the material and the substrate [36]. Pos(G) in thicker samples linearly increase up to ~ 1582 cm^−1^ in the defect-free set and ~ 1580 cm^−1^ in the defective set at 300 K, which means that it is increasingly insensitive to substrate effects as graphene layers become thicker. Meanwhile, FWHM(G) significantly sharpens down to ~ 9.2 cm^−1^ in defect-free 1LG and ~ 10.9 cm^−1^ in defective 1LG in variable temperature experiments although FWHM(G) of pristine graphene is ~ 13 cm^−1^ in room temperature measurements. The possible reason is the blockage of the phonon decay into electron-hole pairs [37] due to the dielectric effect of the substrate in the thinner graphene layer. Finally, D’ phonon can be considered as a nontrivial prototype to study the temperature effect of defective graphene materials based on the following reasons: (1) additional Raman modes can be observed in disordered graphene samples, e.g., the so-called D and D’ modes. Although these modes cannot be attributed to the vibration mode from defects themselves, they correspond to phonons with the breaking of momentum conservation [38] because of the presence of defects in the sample. Their T-dependent behaviors can reflect the contribution from EPC due to the lattice change in defective samples. (2) The relationship between G peak and D’ mode is both interrelated and competitive because there is the related electron-phonon interaction in G and D’ phonon because their frequency and linewidth depend on the same conical electronic band structure in the region near the K point [39]. (3) D phonon is another typical spectral feature in defective graphene samples. However, the D band becomes broad and complex with the increase of graphene layers along the c-axis due to an inter-valley process connecting two conical electronic band structures around inequivalent K and K’ points [40]. (4) More calculation is needed to explain the T-dependent behavior of D’ mode, which is beyond the scope of this work.

## Conclusion

In this paper, vacancies were uniformly introduced into carbon structures by ion C+ bombardment and characterized by Raman mappings of *I*(G). The T-dependent phonon properties of G peak and D’ band in defective 1LG and FLGs were measured by Raman spectrometer combined with a Linkam cryostat, with defect-free samples as a contrast. At temperatures from 78 to 318 K, defects lead to the increase of negative temperature coefficient of G peak due to the lattice change. D’ mode as a Raman signature for disorder is both interrelated and competitive with G peak under the defect-phonon interaction. The temperature coefficient of D’ band is almost identical with G peak. However, Pos(D’) increases simultaneously as FWHM(D’) decreases with increasing layers, contrary to G peak. In conclusion, the defects in graphene structure by ion C+ bombardment induce a large change of T-dependent properties of phonons; therefore, they have an influence on the physical properties of graphene systems. The introduction of foreign atoms into the hexagonal carbon networks has been a hot topic nowadays for an effective tool for tailoring the intrinsic properties of graphene systems. The corresponding properties should be thoroughly investigated in the future.

## Supplementary information

**Additional file 1:** Supplementary Materials

## Data Availability

Graphene flakes were obtained by micromechanical cleavage of bulk graphite crystals (2D semiconductors Inc.) on SiO_2_/Si substrate with SiO_2_ thickness as 89 nm. Their layer number (*N*) was estimated by Raman measurements of the Si intensity ratio between the Si peak (*I*(Si_G_)) from SiO_2_/Si substrate overlying graphene flakes and the Si peak (*I*(Si_0_)) from bare SiO_2_/Si substrate. Vacancies were introduced intentionally by ion C+ bombardment for one set of samples (called the defective set), with the defect-free set as a contrast. The low energy C+ ions bombarded perpendicularly to the sample surface at room temperature which was performed using an LC-4 type system with the dose and kinetic energy of 2 × 10^13^ cm^−2^ and 80 keV, respectively. The Raman mappings were measured at room temperature in back-scattering with a HR Evolution micro-Raman system, equipped with the unique SWIFT™ CCD, a × 100 objective lens (NA = 0.90). An 1800 g/mm grating resulted in a 0.5-cm^−1^ spectral resolution. The laser excitation of 532 nm was used. A laser power below 2 mW was used to avoid sample heating. Mapping measurements were performed using a motorized stage. The *xy* coordinates of each point were previously set in order to find the optimized focus. Mapping images were constructed for each *xy* coordinate by taking 100 points on the surface of a sample with a 10 × 10 equally spaced array of probing points. In all cases *x*, *y* step was 0.5 μm. The T-dependent Raman spectra were measured in back-scattering with a HR Evolution micro-Raman system, equipped with the unique SWIFT™ CCD. The samples were mounted on an in-house-made sample holder consisting of a thin copper disk with a central pillar and a 500-μm diameter hole. Measurements were carried out in a liquid nitrogen (LN_2_) cooled low-temperature Linkam stage equipped with a temperature controller. The programmable cool-stage THMS600 (Linkam Scientific Instruments) covers the temperature range from 78 to 318 K in a N_2_ gas environment. The Linkam instrument has a temperature stability of ± 0.1 K. Using a grating with a groove density of 1800 g/mm, the achieved spectral resolution was 0.5 cm^−1^. A long working distance × 50 objective lens (NA = 0.45) was used, achieving a spatial resolution better than 1 μm. All spectra were excited with a 532-nm laser. During all the measurements, laser power has been kept low enough to prevent any sample heating. The integration time of 20 s was adopted to ensure a good signal-to-noise ratio. The T-dependence of Raman modes was measured in the range from 78 to 318 K and recorded at 10 K intervals, for the defect-free and defective sets.

## Abbreviations

1LG: Monolayer graphene
FLGs: Few-layer graphenes
T-dependent: Temperature-dependent
HOPG: Highly oriented pyrolytic graphite
*N*: Layer number
*L*_D_: The average distance between nearest defects
LN_2_: ,Liquid nitrogen
Pos(G): G peak position
FWHM(G): Full width at half maximum of G peak
EPC: Electron-phonon coupling
