# Supplementary material for: Temperature Dependence of G and D’ Phonons in Monolayer to Few-Layer Graphene with Vacancies
Source: Nanoscale Res Lett. 2020 Sep 30;15:189. doi: 10.1186/s11671-020-03414-w (PMC7527399; doi:10.1186/s11671-020-03414-w)
Supplement: Supplementary file 1 — Additional file 1: Supplementary Materials [file 11671_2020_3414_MOESM1_ESM.docx]

Supplementary Materials

In our experiments, defects were introduced intentionally into sets of graphene flakes by ion implantation. In ion implantation, the amount of implanted C atoms is controlled by the implanted dose. C+ irradiation was performed using an LC-4 type system with the dose and kinetic energy of 2x10^13^ cm^-2^ and 80 keV, respectively. The low energy C cluster ions are implanted perpendicularly to sample surface at room temperature. We prepared multiple samples with different layers, some of whom were taken for ion (C+) bombardment. Because atomic force microscopy (AFM) has been widely used to observe the morphology of nanomaterials, we took an AFM measurement for C+ bombarded 1LG by the tapping mode to minimize sample damage caused by the pin. Fig. f1(a) shows the optical image of C+ bombarded 1LG on the SiO_2_/Si substrate. Fig. f1(b) is the larger version of the optical image of the black rectangle highlighted in Fig. f1(a). Fig. f1(c) is the AFM image of the black rectangle highlighted in Fig. f1(a). We did not see obvious defects on the sample surface. In other words, we found no significant changes in the surface morphology of the ion implanted sample. The possible reason is that the size of the defects in the sample is very small and it is difficult to distinguish them in the AFM images. The ion bombardment energy we chose is relatively low in order to uniformly introduce defects into graphene samples with only some atomic layers thickness. However, confocal micro-Raman spectroscopy, a nondestructive, optical technique has been successfully used to extract defects properties of thin films. Raman spectra were measured in the range of 1250-1650 cm^-1^. After the C+ irradiation, the D band at ∼1350 cm^-1^ and D’ band at ∼1610 cm^-1^ appear in the Raman spectra of the C+ bombarded 1LG, as depicted in Fig. f2(a), meaning that this set became defective. After ion C+ bombardment, vacancies were introduced in graphene layers. Vacancies are one kind of point defects. Point defects can be characterized by the average distance between nearest defects (L_D_). The protocol is based on the intensity ratio between the disorder-induced D band and the G band, i.e., I(D)/I(G), to identify the concentrations of point defects. To avoid the influence of peak broadening and the wavelength dependence of the peak intensity, the integrated intensity (peak area) ratio was used. In the low defect density stage, I(D)/I(G) increases. Point defects are usually quantified using the well-known Tuinstra-Koenig relation I(D)/I(G)=C(k)/L_D_ (J Chem Phys 53: 1126, 1970), where the proportionality constant C(k) depends on the excitation laser wavelength, as depicted in Fig. f2(b). We can calculate the defect distribution L_D_ is about 4-6 nm in C+ bombarded 1LG and is basically homogeneous over almost the entire sheet. The Raman mapping spectra were measured in back-scattering with a HR Evolution micro-Raman system using a 532nm laser. Fig. f2(c) shows the optical image of C+ bombarded 1LG on the SiO_2_/Si substrate. Fig. f2(d) is the Raman mapping spectra of the black rectangle highlighted in Fig. f2(c). Fig. f2(e) is the calculated defect distribution L_D_ of the black rectangle highlighted in Fig. f2(c).

**Figure f1.** Fig. (a) shows the optical image of C+ bombarded 1LG on the SiO_2_/Si substrate. Fig. (b) is the larger version of the optical image of the black rectangle highlighted in Fig. (a). Fig. (c) is the AFM image of the black rectangle highlighted in Fig. (a).

**Figure f2.** Fig. (a) shows Raman spectra of C+ bombarded 1LG in the range of 1250-1650 cm^-1^ with the defect-free 1LG for comparison. Fig. (b) shows the well-known Tuinstra-Koenig relation I(D)/I(G)=C(k)/L_D_ (J Chem Phys 53: 1126, 1970). Fig. (c) shows the optical image of C+ bombarded 1LG on the SiO_2_/Si substrate. Fig. (d) is the Raman mapping spectra of the black rectangle highlighted in Fig. (c). Fig. (e) is the calculated defect distribution L_D_ of the black rectangle highlighted in Fig. (c).

After ion C+ bombardment, additional Raman modes can be observed, e.g., the so-called D and D’ modes. Raman spectra of C+ bombarded 1LG, 2LG, and 3LG were measured at room temperature in the range of 1250-1650 cm^-1^ with D band, G band and D’ band contained, as depicted in Fig. f3(a). The Raman mapping spectra were also measured at room temperature in back-scattering with a HR Evolution micro-Raman system. Because I(D)/I(G) shows the linear dependence at low defect concentration as reported (I(D)/I(G)=C(k)/L_D_), we can use the distribution of I(D) or I(G) over the entire sheet to determine the uniformity of vacancies. We have shown Raman mapping of I(G) for C+ bombarded 1LG, 2LG, and 3LG in Figure 2 of the manuscript. The Raman mappings of I(D) and I(G) simultaneously for C+ bombarded 1LG, 2LG, and 3LG were shown as follows, as depicted in Fig. f3(b)-(d). The color of I(D) mappings in almost all samples are basically homogeneous over the entire sheet to determine the uniformity of vacancies.

In addition, I(G) is sensitive to the number of defects at low defect concentrations in graphene systems because G peak arises from the in-plane C-C bond stretching of all pairs of sp^2^ atoms in both rings and chains. Moreover, G peak is a phonon originating from a normal first-order Raman scattering process in graphene systems, its intensity can be enhanced because of the resonance process due to the excitation energy matching the transition from a valence band to a conduction band. The I(G) mappings can be used in all samples before and after ion C+ bombardment to determine the uniformity of atomic structure of graphene layers. However, the D feature becomes broaden and complex in FLGs similar to 2D band. I(D) is much larger than I(G) in C+ bombarded 1LG, but is much lower than I(G) in C+ bombarded 2LG and 3LG. Thus, we used I(G) mappings to identify degree and uniformity of defects in the manuscript.

**Figure f3.** Fig. (a) shows Raman spectra of C+ bombarded 1LG, 2LG, and 3LG measured at room temperature in the range of 1250-1650 cm^-1^ with D band, G band and D’ band contained. Fig. (b)-(d) show the Raman mappings of I(D) and I(G) simultaneously for C+ bombarded 1LG, 2LG, and 3LG.
